# A global dataset of plant available and unavailable phosphorus in natural soils derived by Hedley method

**DOI:** 10.1038/sdata.2018.166

**Published:** 2018-08-21

**Authors:** Enqing Hou, Xiang Tan, Marijke Heenan, Dazhi Wen

**Affiliations:** 1 Key Laboratory of Vegetation Restoration and Management of Degraded Ecosystems, South China Botanical Garden, Chinese Academy of Sciences, Guangzhou 510650, China; 2 Key Laboratory of Aquatic Botany and Watershed Ecology, Wuhan Botanical Garden, The Chinese Academy of Sciences, Wuhan 430074, China; 3 Department of Science, Information Technology and Innovation, Queensland Government, Dutton Park, Brisbane, Queens Land 4102, Australia; 4 Guangdong Provincial Key Laboratory of Applied Botany, South China Botanical Garden, Chinese Academy of Sciences, Guangzhou 510650, China

**Keywords:** Element cycles, Ecosystem ecology

## Abstract

Soil phosphorus (P) fractions are critical for understanding soil P dynamics and availability. This paper provides a global dataset of soil P fractions separated by the Hedley method. The dataset also includes key environmental factors associated with soil P dynamics and availability, including climate factors, vegetation, soil and parent material types, soil age, and soil physiochemical properties such as particle size, bulk density, pH in water, organic carbon, total nitrogen, and extractable iron and aluminium concentrations. This dataset includes measures of Hedley P fractions of 802 soil samples and was gathered through a literature survey of 99 published studies. Plant availability of each soil P fraction was noted. We anticipate that the global dataset will provide valuable information for studying soil P dynamics and availability, and it will be fused into earth system models to better predict how terrestrial ecosystems will respond to global environmental changes.

## Background & Summary

Phosphorus (P) is a key limiting nutrient of plant growth and soil microbial activity. Atmospheric P input is extremely low (<0.1 kg P ha yr^−1^) in most global land areas^1^. Therefore, soil is typically the major source of P to plants and soil microbes in terrestrial ecosystems^2,3^. Soil P supply or availability plays a vital role in determining the structures, functions, and processes of terrestrial ecosystems^2,4–7^. For example, insufficient soil P supply accounts for P limitation on plant production in terrestrial ecosystems worldwide^8,9^. The growth of different plant or soil microbe species varied in their dependencies on soil P supply^6^. Soil P supply is, therefore, an important driver of the composition and diversity of plant and soil microbial communities^6,10^. Since low soil P supply can limit soil microbial activity^11^, soil P supply also regulates litter decomposition and soil organic C dynamics^7,12,13^. Improved understanding of soil P dynamics and availability is critical for better understanding of nutrient regulation on key ecosystem properties in terrestrial ecosystems.

**Figure 1:**
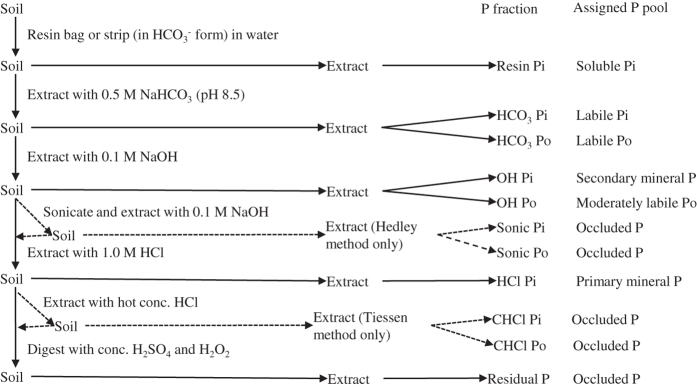
A flow chart of soil P fractionation. The flow chart follows the procedures of Hedley, *et al*.^17^ and Tiessen and Moir^24^. Microbial biomass P estimates in Hedley, *et al*.^17^ had not been in common use and therefore was not included in the flow chart. “Sonicate and extract with 0.1 M NaOH” was available only in the procedure of Hedley, *et al*.^17^; “Extract with hot concentrated HCl” was available only in the procedure of Tiessen and Moir^24^. Soil P pools were assigned according to previous studies^25,30–33,38^.

Total P stock of soil is always large compared to that of vegetation P stock^14,15^. However, soil P supply is rarely adequate in meeting the P demands of plants in terrestrial ecosystems^9,16^. This is largely due to multiple forms of P existing in the soils, which differ in their availability for plant uptake across time scales^17,18^. Soil P availability is controlled by sorption/desorption, precipitation/dissolution, immobilization/mineralization, weathering, and solid-phase P transformations such as solid-phase diffusion or penetration, recrystallization, and migration in aggregates^2,19–22^. A detailed investigation of P dynamics and bioavailability requires the separation and identification of different forms of P in soils^23^. The method of Hedley, *et al*.^17^ and its modification by Tiessen and Moir^24^ (Fig. 1) are the most commonly used procedures for the sequential fractionation of P in soils. These procedures enable the characterization of different inorganic P (Pi) and organic P (Po) fractions based on their solubility. Investigating changes in these soil P fractions along environmental (e.g. climate) gradients can provide important insights into environmental controls over long-term soil P dynamics and availability^25–30^.

Several studies have summarized data of Hedley P fractions in worldwide natural soils^25,31,32^. In 1995, Cross and Schlesinger^25^ compiled Hedley P fractions of 88 natural, unfertilized or uncultivated soil samples worldwide from 16 published studies. Based on this dataset, the authors explored changes in soil P dynamics across a soil weathered gradient. In 2011, Yang and Post^32^ updated this global dataset to 178 soil samples from 28 published studies, to further explore soil P dynamics along a pedogenesis gradient. Based on data of the 178 soil samples, Yang *et al*.^33^ provided the first spatially explicit estimates of different forms of P in 0–50 cm mineral soils at the global scale in 2013. These estimates (usually only labile P, sometimes in combination with a soil P dynamics model) have been used as an index of soil P supply in a few earth system models to diagnose P limitation on plant growth at the global scale^34–37^. However, a large uncertainty in P limitation has been found in these studies, largely due to a deficiency in the number of soil P fraction measurements^34–37^. In 2015, Hou *et al*.^31^ updated this global dataset to 626 soil samples from 85 published studies, to test hypothetical models of soil P transformations. In 2017, Hou *et al*.^30^ further updated this global dataset to 802 soil samples from 98 published studies and one unpublished study (i.e., ‘Hou *et al*., unpublished’), to examine climate effects on soil P cycle and availability. The ‘Hou et al., unpublished’ was published recently^29^. Therefore, our global database now included 802 soil samples from 99 published studies.

Here, we provide a detailed description of the global database for use in further studies. Besides data of Hedley P fractions, the dataset also contained various environmental factors that potentially affect soil P dynamics and availability, such as mean annual temperature (MAT), mean annual precipitation (MAP), soil pH, organic C and extractable iron (Fe) and aluminium (Al) concentrations. This global dataset is expected to permit detailed analysis of the controls of environmental factors, e.g. climate and soil pH, on soil P dynamics and availability^30^. It can also be incorporated into earth system models^7,34–37^, to constrain the prediction of climate effect on primary productivity and C storage in global terrestrial ecosystems. Our dataset is about four times as large as that of Yang and Post^32^ in terms of both soil number and publication number. In particular, our dataset included much more data from Asia (269), Africa (40), and Oceania (20) which were underrepresented previously (3, 4, and 0, respectively).

## Methods

### Soil P fractionation procedure

Hedley P fractionation procedure and its modifications are designed to indicate soil P pools^17,24,25^. The terminology of Hedley P fractions have been different in different studies^25^. Some consensus, however, has been reached during the last three decades^25^. In general, the resin Pi fraction represents the soil solution or soluble Pi pool, which can be immediately accessed by plants^31,38^. If depleted, the soluble Pi will be replenished by solid-phase Pi pools via desorption, dissolution, or solid-phase P transformation and by solid-phase Po pools via mineralization^20,22^. The HCO_3_ Pi fraction is considered a labile Pi pool that can be released by ligand exchange with the bicarbonate ion; this Pi pool is available to plants and persists for only short periods, e.g., a growing season^25,32^. The HCO_3_ Po fraction represents a labile Po pool that can be utilized by plants after being mineralized^22^. The OH P (Pi and Po) fractions index moderately labile P (Pi and Po) pools that are bound with amorphous and some crystalline Al and Fe^22^, with low availability to plants^22,25^. The dilute HCl Pi fraction indexes a primary mineral P pool that is bound with calcium (Ca) and that can be utilized by plants after it is released by weathering^25,31^. Other P fractions such as residual P (Fig. 1) usually indicate the occluded P pool that is least available to plants due to their particularly low solubility^23,24,39^.

A summarized description of the procedures of Hedley, *et al*.^17^ and Tiessen and Moir^24^ was shown in Fig. 1 and also as follows:

1. Resin extract: weigh 0.5 g air-dried soil into a 50 ml centrifuge tube, add 2 resin strips (in HCO_3_^−^ form) + 30 ml deionized water, and shake 16 h. Remove resin strips from the tube. Then place resin strips in a clean 50 ml centrifuge tube, add 20 ml 0.5 M HCl, set aside for 1 h, and collect the 0.5 M HCl extract for P measurement. Centrifuge the tube with soil suspension, discard the supernatant, and keep the soil for further extractions.

(In the Hedley procedure, duplicate 0.5 g air-dried soils are prepared; one of the soil samples is added with 1 ml CHCl_3_ for the determination of soil microbial biomass P. However, this procedure has rarely been used by later studies. In our database, only 3 of the 41 measurements of soil microbial biomass P were determined according to the Hedley procedure.)

2. HCO_3_^−^ extract: add 30 ml 0.5 M NaHCO_3_ at pH 8.5 to the soil, shake 16 h, centrifuge, filter (<0.45 mm) and collect the supernatant for P measurement, and keep the soil for further extractions.

3. OH^−^ extract: add 30 ml 0.1 M NaOH to the soil, shake 16 h, centrifuge; filter (<0.45 mm) and collect the supernatant for P measurement, and keep the soil for further extractions.

(In the Hedley procedure, a second extraction with 30 ml 0.1 M NaOH and sonication is used after step 3. This procedure has been, however, usually absent in later studies, e.g. Paré and Bernier^40^.)

4. HCl extract: add 30 ml 0.1 M HCl to the soil, shake 16 h, centrifuge; filter (<0.45 mm) and collect the supernatant for P measurement, and keep the soil for the following digestion.

(An important modification of the Hedley procedure by Tiessen and Moir^24^ is removing the second OH^−^ extraction in the Hedley procedure but adding an extraction of 10 ml hot (at 80 °C) concentrated HCl after step 4.)

5. Residual fraction: Digest the soil with 5 ml concentrated H_2_SO_4_ and H_2_O_2_, filter (<0.45 mm) and collect the solution for P measurement.

Inorganic P in all the above extracts are determined using the molybdate blue method^41^. Organic P of the HCO_3_^−^ extract, OH^−^ extract, and second OH^−^ extract or hot conc. HCl extract are calculated as the difference between total P determined after persulphate digestion^42^ and inorganic P. Phosphorus in the resin and HCl extracts are usually considered to be totally in inorganic form^22^.

### Literature search

**Figure 2:**
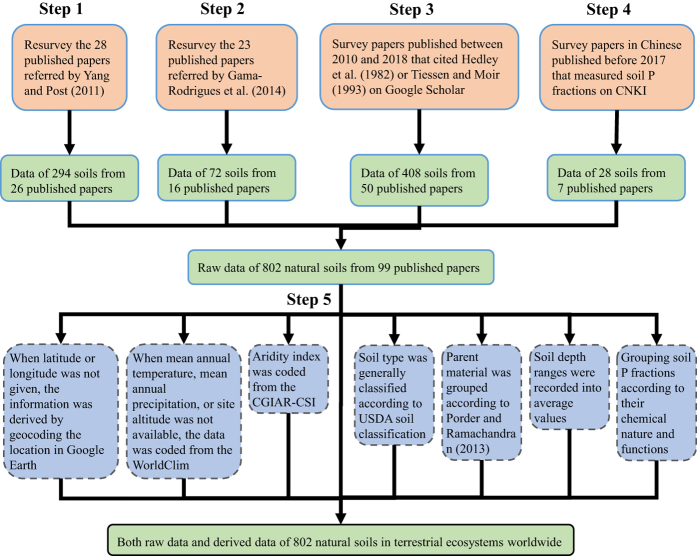
Flowchart of the steps in the literature search and data management. Steps 1–4 indicate the four steps in the literature search. Step 5 indicates the management of some of the raw data from the literature.

In general, we compiled a database of soil P fractions by surveying the peer-reviewed, published research that used the sequential fractionation techniques developed by Hedley, *et al*.^17^ and modified by Tiessen and Moir^24^. Our survey was restricted to studies of unfertilized, uncultivated, and (semi-) natural soils. We defined (semi-) natural soils as those in sites with primary vegetation or with a stand age greater than 10 years for forests, which is consistent with a study of Hedley P fractions in tropical soils^26^. Our survey included papers published as recently as April 2017 except one study published in 2018 (ref. 29). We collected data of Hedley P fractions in soils at all reported depths in various habits/landscapes (e.g. forest lands, grasslands, and savanna lands). There is no additional criteria for studies to be included. In general, we collected data in four steps, as summarized in Fig. 2 and described in detail as follows:

Yang and Post^32^ comprehensively surveyed peer-reviewed published research that reported values of Hedley P fractions (following the procedure of Hedley, *et al*.^17^ or Tiessen and Moir^24^) in worldwide unfertilized, uncultivated, and natural soils before 2010 (data of 178 soil samples from 28 studies). Gama-Rodrigues, *et al*.^26^ collected data of Hedley P fractions in tropical soils using the similar method (data of 81 soil samples from 23 studies). To simplify our survey of early studies (published before 2010), we resurveyed papers listed in these studies. It is noted that these two studies collected data of only surface soils (mostly at a mineral depth of 0–15 cm). Since deep (>15 cm) soils are important components of terrestrial ecosystems that can intensively interact with surface soils^43^, we collected data of Hedley P fractions in soils at all reported depths. (1) The resurvey of published papers referred by Yang and Post^32^ resulted in a dataset of 294 soil samples from 26 published papers. (2) The resurvey of published papers referred by Gama-Rodrigues, *et al*.^26^ resulted in a dataset of 72 soil samples from 16 published papers.

And then, (3) we comprehensively surveyed all peer-reviewed papers that cited Hedley, *et al*.^17^ or Tiessen and Moir^24^ and published during the period of 2010 to April, 2017 on Google Scholar. An exception was a study published in 2018 (ref. 29). We did the survey by reading the title, abstract, and/or the full text of each of the papers. During this literature survey, data from 408 additional soil samples in 50 additional studies were collected. Finally, (4) we comprehensively surveyed peer-reviewed papers published in Chinese before April, 2017, using keywords of “soil” and “phosphorus fraction” (in Chinese) on the website of Chinese National Knowledge Infrastructure (CNKI, website: http://www.cnki.net/). Since the database of CNKI is not well linked to English journals, we surveyed papers in Chinese using keywords rather than tracing the citations of Hedley, *et al*.^17^ or Tiessen and Moir ^24^. During this literature surveying, data from 28 soil samples in 7 studies were collected.

In total, we collected data of Hedley P fractions in 802 natural soil samples from 99 published studies. In our database, all data were collected at the plot scale. For data with sample replicates in the same plots, the average values per plot were calculated and used. Typically, there is no analytical duplicate for Hedley P fractionation. Some descriptions and analyses of this database or its sub-databases were given in previous studies^30,31^.

## Data Records

The database file is in xlsx format and the reference list in pdf format. Both files were archived in PANGAEA (Data Citation 1). Blank denotes missing data. The database included both raw data from the published studies and the data derived from global maps or recalculated by the authors (Fig. 2).

Raw data compiled from the published studies are listed as follows in the format of ‘variable name (location in the database; unit): variable description’:

Code (column 1 of the database (C1 in abbreviation, the same below)): label of the soil sample.

Reference (C2): the referred studies

Country (C3): the country where the study site located

Site (C4): name of the site where the study performed

Latitude (C5; −43.25 to 69.35): in decimal degrees

Longitude (C6; −117.86 to 171.58): in decimal degrees

MAT (C7; °C): mean annual temperature

MAP (C8; mm yr^−1^): Mean annual precipitation

Elevation (C9; m, a.s.l.)

Slope (C10; ^o^): site slope with unit of degree or percentage

Vegetation type (C11): as described in the referred study, mostly of forest and grass.

Stand age (C12; yr): stand age of forest ecosystems. It is either a specific stand age (e.g. 20) or a description such as ‘Native’ or ’Primary’ forest.

Parent materials (C13): as described in the referred study.

Soil type (C14): mostly classified according to the soil classification system of the country where the study performed.

Soil classification system (C15): soil classification systems used to define soil types in the referred studies.

Soil age (C16; yr)

Soil note (C17): label of the soil, as described in the referred studies

Soil horizon (C18): either a range of soil depth (e.g. 0–15 cm) or a description of soil horizon (e.g. A horizon)

Water Pi (C19; mg kg^−1^): some studies modified Hedley procedure by replacing resin extraction with water or KCl extraction, e.g. Vu, *et al*.^44^.

Resin Pi (C20; mg kg^−1^)

HCO_3_ Pi (C21; mg kg^−1^)

HCO_3_ Pi2 (C22; mg kg^−1^): some studies modified Hedley procedure by extracting P from soils firstly with 0.5 M NaHCO_3_ at pH 8.5 (i.e. without a resin extract), e.g., Lilienfein, *et al*.^45^, or reported only the sum of, but not the individual values of, the HCO_3_ Pi fraction and the resin (or water) Pi fraction, e.g., Satti, *et al*.^46^.

HCO_3_ Po (C23; mg kg^−1^)

OH Pi (C24; mg kg^−1^)

OH Po (C25; mg kg^−1^)

HCl Pi (C26; mg kg^−1^)

Sonic Pi (C27; mg kg^−1^)

Sonic Po (C28; mg kg^−1^)

CHCl Pi (C29; mg kg^−1^)

CHCl Po (C30; mg kg^−1^)

Residual P (C31; mg kg^−1^)

Total Po (C32; mg kg^−1^): total organic P measured separately

Sum of P fractions (C33; mg kg^−1^): sum of all Hedley P fractions, generally equal to soil total P

Soil total P (C34; mg kg^−1^): measured separately using a digestion method^47^

Comment (C35): Notes about soil P fractions

pH (C36): soil pH in water

TOC (C37; %): soil total organic carbon

TN (C38; %): soil total nitrogen

DCB_Al (C39; mg kg^−1^): dithionite-citrate-bicarbonate extractable soil Al^48^

DCB_Fe (C40; mg kg^−1^): dithionite-citrate-bicarbonate extractable soil Fe^48^

Oxa_Al (C41; mg kg^−1^): oxalate extractable soil Al^48^

Oxa_Fe (C42; mg kg^−1^): oxalate extractable soil Fe^48^

DBD (C43; g cm^−3^): soil bulk density

Texture (C44): soil texture as described in the referred studies (e.g. coarse loamy)

Sand (C45; %): soil sand content (diameter between 0.05 mm and 2.00 mm)

Silt (C46; %): soil silt content (diameter between 0.002 mm and 0.05 mm)

Clay (C47; %): soil clay content (diameter<0.002 mm)

Method comment (C48): method for the determination of soil particle size, mostly with the pipette or hydrometer method^49^.

MBP (C49; mg kg^−1^): soil microbial biomass P; a total of 41 values, 38 of which had been separately determined by a fumigation-extraction method^50^ rather than as a fraction of the Hedley procedure^17^

Reorganized data by the authors as follows:

Latitude2 (C50) and Longitude2 (C51): in decimal degrees. In cases where the referred studies did not report the latitude or longitude of the measurement, the approximate latitude or longitude were derived by geocoding site name in Google Earth 7.0

MAT2 (C52); MAP2 (C53), Elevation2 (C56): In cases where the referenced studies did not report MAT, MAP, or elevation, the values were derived from WorldClim^51^ using site geographic location (i.e., latitude and longitude)

Aridity index (C54): aridity index that was derived from CGIAR-CSI^52^ using site geographic location

Soil type2 (C56): soil type classified according to the USDA soil classification system^53^. For soil types that were initially not classified according to the USDA soil classification system, they were reclassified according to the USDA soil classification system by referring the descriptions in published studies through searching the soil type described by the referred study (e.g. ‘Ferric Acrisol’) and ‘USDA’ in Google Scholar.

Parent material2 (C57): parent materials grouped mainly according to Porder and Ramachandran^54^, except glacial till and volcanic ash which were treated as two separate groups

Vegetation type2 (C58): vegetation type grouped into seven groups, i.e. forest, shrub, savanna, grass, meadow, pasture, tundra

Slope2 (C59; ^o^): soil slope with unit expressed in degree. Site slope expressed in percentage was transformed to data in degree

Depth_soil (C60; m): soil depth ranges were recoded into average value (e.g., ‘0–15 cm depth’ was recoded as ‘0.075’)

Depth_note (C61): soils of organic layer or mineral layer classified according to soil genesis

Code (C62): 0 indicates organic layer; 1 indicates averaged soil depth between 0 and 10 cm; 2 indicates averaged soil depth between 10 cm and 20 cm; 3 indicates averaged soil depth >20 cm; 4 indicates mineral soil at unknown soil depth; 5 indicates unknown soil horizon

Total P2 (C63): soil total P; mostly of the sum of P fractions; if sum of P fractions was not given or can't be calculated from available data, separately measured soil total P was used

Labile Pi or available P (C64; mg kg^−1^): sum of HCO_3_ Pi (C20) and resin Pi (C19)/water Pi (C18), or HCO_3_ Pi2 (C21)

Organic P (C65; mg kg^−1^): calculated as the sum of HCO_3_ Po (C22) and OH Po (C24)

Primary P (C66; mg kg^−1^): primary mineral P, which was HCl Pi (C25)

Secondary P (C67; mg kg^−1^): secondary mineral P, which was OH Pi (C23)

Occluded P (C68; mg kg^−1^): the sum of residual P (C30), sonic Pi (C26), and sonic Po (C27) obtained by the Hedley procedure; the sum of residual P (C30), CHCl Pi (C28), and CHCl Po (C29) obtained by the Tiessen and Moir procedure; or the difference between total P (C62) and the sum of resin Pi (C19), HCO_3_ Pi (C20) and HCO_3_ Po (C22), OH Pi (C23) and OH Po (C24), and HCl Pi (C25) obtained by the studies in which neither a second OH^−^ extract nor a hot conc. HCl extract was included.

### Data overview

**Figure 3:**
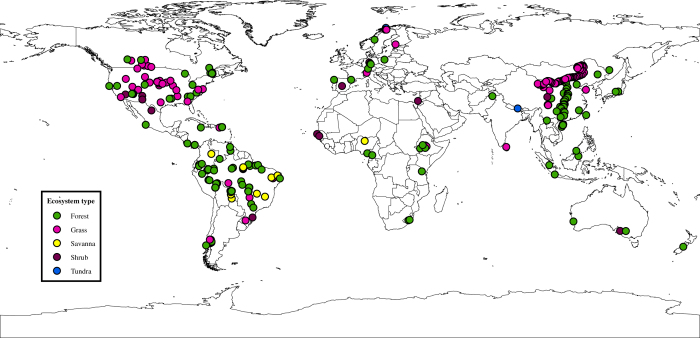
Distribution of soil sample locations. Soil sample locations contain multiple data entries.

Sites in our database were located on all continents except Antarctica (Fig. 3). The database spanned over 112^o^ in latitude (43.3^o^S–69.4^o^N; Table 1 and Fig. 3). MAT ranged from -7.1°C to 29.0°C. MAP ranged from 31 to 6000 mm yr^−1^. Elevation ranged from 11 m to 4235 m. Average soil depth ranged from 1 cm to 450 cm. Soil pH in water ranged from 3.2 to 9.5. Soil P fractions generally varied over three orders (Table 1).

Among the 802 soil samples, values for the sonic Pi, sonic Po, CHCl Pi, and CHCl Po fractions were missing for about 85% (84.2–85.3%) of the samples (Table 1). This was partly because studies that used the procedure of Hedley, *et al*.^17^ did not have values of the CHCl Pi, and CHCl Po fractions, and studies that used the procedure of Tiessen and Moir^24^ did not have values of the sonic Pi and sonic Po fractions. Moreover, some studies, e.g. Vu, *et al*.^44^, modified the procedure of Hedley, *et al*.^17^ by omitting the extract of second 0.1 M NaOH and sonication. Values for the resin Pi and HCO_3_ Pi fractions were missing for 32.3 and 26.1%, respectively, of the samples. Data were missing for these two fractions partly because the resin Pi fraction was not separated from the HCO_3_ Pi fraction (e.g., Lilienfein, *et al*.^45^) (Table 1). Data were missing for the resin Pi fraction also because resin was replaced by deionized water or KCl solution to extract the most soluble P pool in some studies (e.g., Vu, *et al*.^44^) (Table 1). For the other P fractions, data were missing mainly because the specific P fraction value (Pi or Po) was not indicated (e.g., HCO_3_ Po in Garcia-Montiel, *et al*.^55^). For other parameters in the database, data were missing either because the values were not indicated or because the measurement method did not fulfill our survey requirements. Missing data would not hinder the use of our dataset by most researchers, as shown in our previous studies^30,31^. Missing data may be either deleted or filled using multiple imputation methods before statistical analyses. The dataset may be also analyzed with statistical methods that can deal with missing data such as boosting regression tree.

## Technical Validation

A test of relationships between data of climate and altitude reported in the referred studies and those derived from WorldClim (mean annual temperature: r=0.95, *P*<0.001, n=407; mean annual precipitation: r=0.85, *P*<0.001, n=459; elevation: r=0.88, *P*<0.001, n=328) indicates that the derived data from WorldClim were generally reliable for our study sites.

## Usage Notes

The availability of P in soil to plant is strongly time-dependent^18,22,56^. Definition of the availability of a soil P fraction to plant is also time-dependent^22,25^. Here, we provide some advices for the definition of plant available and unavailable P in soils derived by Hedley fractionation, as summarized in some previous studies^22,25,31^. The resin Pi and HCO_3_ Pi fractions function similarly in soils^25,32^, with turnover times likely of a few days^18,57^; therefore the two P fractions can be always defined as plant available P^25,32^. There is probably a continuum of solubility among the resin Pi, HCO_3_ Pi, and OH Pi fractions^31,58^. However, the OH Pi fraction turnovers more slowly than the resin Pi and HCO_3_ Pi fractions, which have a likely turnover time of months^18^. Therefore, the OH Pi fraction may be available to plant in months or over longer terms^18,56^. Similar to the Pi fractions, there is also a continuum of solubility between the HCO_3_ Po and OH Po fractions, with the former having a somewhat faster turnover than the latter one^22,31^. The HCO_3_ Po fraction may be considered as soil available P in weeks or longer terms^25^; while the OH Po fraction could be also available to plants in seasons or longer terms^56,59^. The HCl Pi fraction is typically slow-changing^60^ and can be available to plants in decades or longer terms^31,61^. The sonic Pi, sonic Po, conc. HCl Pi, conc. HCl Po, and residual P fractions all turnover slowly in soils^25,59^, but their roles (either as a source or as a sink of soil available P) in controlling soil P availability should be considered in decades or longer terms^31,61^. Finally, it’s noted that the same soil P fraction is not of equal availability to plants in all soils^56^, but is influenced by soil conditions (e.g. weathered extent)^56^, plant species^62^, and environmental conditions (e.g. temperature and precipitation)^30^.

Hedley P fractions are usually grouped according to the similarity of their functions and chemical natures, to simplify statistical analysis and/or facilitate data interpretation. Here, we have several suggestions inline with this. (1) Sum of the resin Pi fraction and the HCO_3_ Pi fraction may be used as an index of labile inorganic P or available P, as frequently used in some previous studies^18,25,30^. This is because resin used for the Hedley P fractionation is typically in HCO_3_^−^ form, which extract P from soil in a similar manner (i.e. through ion exchange) as 0.5 M NaHCO_3_ (pH 8.5)^25^. Functional similarity between the resin Pi fraction and the HCO_3_ Pi fraction was also suggested by the close relationship between them found in previous studies^31^. (2) Sum of the HCO_3_ Po fraction and the OH Po fraction, and also the second OH Po fraction or the CHCl Po fraction if available, may be used as an index of soil organic P. (3) To reconcile the difference in defining the residual P fraction among publications^25^, a measure of occluded P, recalcitrant P, or residual P may be calculated in one of the three following ways^31^: the sum of residual P, sonic Pi, and sonic Po fractions obtained by the Hedley procedure; the sum of residual P, conc. HCl Pi, and conc. HCl Po fractions obtained by the Tiessen and Moir procedure; or the difference between total P and the sum of resin Pi, HCO_3_ Pi and Po, OH Pi and Po, and HCl Pi fractions.

Soils in our database varied largely in their depths, of which half had an average soil depth ≤ 10 cm (Table 1). Biogeochemistry-climate models typically rely on the properties of soils with the same depth (e.g. 0-50 cm)^34–37^. Soil P fractions in our database need to be unified before its usage by biogeochemistry-climate models. One possible way to do this is recalculating the soil P fraction values using the empirical relationships between soil depth and soil P fractions.

A full list of references used to build our database is given in the References section^25,29,40,44,45,46,55,59,63–153^.

## Additional information

**How to cite this article**: Hou, E. *et al*. A global dataset of plant available and unavailable phosphorus in natural soils derived by Hedley method. *Sci. Data* 5:180166 doi: 10.1038/sdata.2018.166 (2018).

**Publisher’s note**: Springer Nature remains neutral with regard to jurisdictional claims in published maps and institutional affiliations.

## Supplementary Material



## Figures and Tables

**Table 1:** A summary of the continuous variables in the global database of Hedley P fractions.

Parameter	N	Missing proportion (%)	Mean	Median	Range	SD	Skewness
Latitude	720	10.2	18.5	24.5	−43.3–69.4	27.1	−0.4
Longitude	719	10.3	2.9	−40.2	−117.9–171.6	87.9	0.3
Elevation (m, a.s.l.)	501	37.5	1220	805	11–4235	1041	1.0
Slope (^o^)	166	79.3	10.6	7.4	0–45.0	11.5	1.1
Mean annual temperature (°C)	604	24.7	14.8	14.7	−7.1–29.0	9.6	−0.2
Mean annual precipitation (mm yr^−1^)	667	16.8	1747	1526	31–6000	1210	0.7
Aridity index	800	0.2	1.3	1.1	0.03–3.92	0.9	1.0
Soil age (yr)	45	94.4	159443	110	12–3000000	535054	4.5
Forest stand age (yr)	77	90.4	58	25	10–300	71	2.1
Soil depth (cm)	684	14.7	18.3	10.0	1–450	31.1	7.4
Resin Pi fraction (mg kg^−1^)	551	31.3	18.9	7.0	<0.1–271	34.6	3.8
HCO3 Pi fraction (mg kg^−1^)	593	26.1	14.8	7.7	<0.1–204	20.9	3.5
HCO3 Po fraction (mg kg^−1^)	710	11.5	26.2	11.2	<0.1–395	39.8	3.7
OH Pi fraction (mg kg^−1^)	751	6.4	40.3	24.7	<0.5–435	47.5	2.8
OH Po fraction (mg kg^−1^)	706	12.0	104.6	52.6	<0.5–910	134.6	2.3
HCl Pi fraction (mg kg^−1^)	709	11.6	86.8	22.7	<0.1–1151	154.7	3.3
Sonic Pi fraction (mg kg^−1^)	120	85.0	14.4	5.2	<0.1–208	28.9	4.1
Sonic Po fraction (mg kg^−1^)	118	85.3	20.7	11.2	<0.1–163	28.6	2.9
CHCl Pi fraction (mg kg^−1^)	125	84.4	64.1	42.9	<0.1–253	54.6	1.2
CHCl Po fraction (mg kg^−1^)	127	84.2	36.8	21.6	<0.1–222	44.8	1.9
Residual P fraction (mg kg^−1^)	717	10.6	155.9	117.0	<0.5–998	153.5	2.1
Soil microbial biomass P (mg kg^−1^)	53	93.4	65.4	51.0	3–230	54.1	0.7
Total organic P (mg kg^−1^)	359	55.2	163.8	81.9	0.4–1176	202.0	2.3
Soil pH	607	24.3	5.6	5.2	3.2–9.5	1.3	0.6
Soil organic C (%)	652	18.7	5.5	2.3	0.02–54.5	8.7	3.1
Soil total N (%)	476	40.6	0.34	0.18	0.002–3.3	0.44	3.0
DCB-Al (g kg^−1^)	89	88.9	2.0	1.0	0.3–18.5	2.8	3.8
DCB-Fe (g kg^−1^)	128	84.0	18.5	9.1	0.4–251	29.2	4.9
Oxalate-Al (g kg^−1^)	136	83.0	3.7	2.5	0.03–25.9	4.5	3.1
Oxalte-Fe (g kg^−1^)	164	79.6	4.5	2.9	0.01–121	10.2	9.4
Soil bulk density (g cm^−3^)	123	84.7	1.2	1.3	0.1–1.8	0.4	−1.1
Soil sand content (%)	332	58.6	47.1	48.8	1–98	28.1	0.0
Soil silt content (%)	312	61.1	27.8	24.4	1–85	18.8	0.7
Soil clay content (%)	386	51.9	26.6	22.0	0.1–91	19.7	1.1
Note: all data are raw values from the referred studies, except aridity index which was derived from CGIAR-CSI.							
^a^For each soil, soil depth range was recoded into an average value (e.g., ‘0–15 cm depth’ was recoded as ‘0.075’).							
^b^In some studies, deionized water, instead of resin, was used to extract the most soluble P pool in soil.							
^c^In some studies, 0.5 M NaHCO_3_ at pH 8.5 was used as the first reagent to extract P from soil (i.e., without an resin extract) or reported only the sum of but not the individual values of the HCO_3_ Pi fraction and the resin (or water) Pi fraction.							
